# Were camptodactyly and boutonniere deformity considered pathological in late fifteenth century Italy? Evidence from the sculptures of Francesco di Simone Ferrucci (1437–1493)

**DOI:** 10.1007/s00296-023-05434-y

**Published:** 2023-09-01

**Authors:** W. R. Albury, G. M. Weisz

**Affiliations:** 1 School of Humanities and Languages, University of New South Wales, Sydney, NSW 2052 Australia; 2 School of Humanities, Arts and Social Sciences, University of New England, Armidale, NSW 2351 Australia

**Keywords:** Medicine in the arts, Hand deformities, acquired, Hand deformities, congenital, Epidemiology, historical, Nosology, historical

## Abstract

Representations of disease in Renaissance paintings have been discussed in medical literature, in the context of historical epidemiology, as potential sources of information about the incidence and appearance of particular conditions in earlier times. The present study seeks to show how Renaissance art can also contribute to historical nosology by casting light on the question of whether particular conditions recognized as abnormal today were understood as pathological in the past. The hands of two Renaissance Madonna figures are examined in sculptures produced by Francesco di Simone Ferrucci (1437–1493). Because the Virgin Mary was considered physically perfect by believers, and because Francesco was a successful producer of devotional sculptures for a wide audience, it is highly probable that any abnormal conditions found in the hands of Madonnas sculpted by him would not have been regarded as pathological at the time. The sculptures examined appear to depict camptodactyly and boutonniere deformity in the hands of Madonna figures. These uncommon conditions are also found in Renaissance artworks that show other individuals of high social status, but their presence in the hands of the Madonna gives the strongest indication that they were not considered pathological, due to religious belief in the Virgin’s physical perfection. Examination of Madonna figures in late fifteenth century Renaissance art can contribute to historical nosology by identifying abnormal conditions that were not regarded as pathological at the time. The examples of such conditions identified in the present study are camptodactyly and boutonniere deformity.

## Introduction/objective

The process of identifying pathologies and anatomical anomalies in older works of art has played a role in medical discussions about the past incidence of particular disorders and possible changes in the presentation of these disorders over time [1–3]. In such discussions much of the debate has centered on the question of whether the apparent pathologies and deformities found in individual artworks are depictions of actual occurrences of these disorders, or are stylistic features introduced by the artist for aesthetic reasons [2, 4, 5].

In the first case, where paintings or statues from earlier times are accepted as depicting actual occurrences of non-physiological conditions, they can provide information that contributes to historical epidemiology by supplementing written records, if any, and paleopathological or other forms of physical evidence. But in the second case, where the anomalies depicted are regarded as stylistic features rather than realistic representations, then it is usually considered that although an error of misdiagnosis has been avoided, the artwork in question has no positive information of medical interest to offer.

It has been argued, for example, that some conditions recognized as abnormal today, such as clinodactyly and camptodactyly of the hand, were often depicted in Renaissance paintings as signs of ‘a certain delicacy and grace’ rather than as diseases or defects [6, 7]. This leaves open, however, the issue of whether the conditions in question were understood as pathological at the time, especially when there is no known written material from the period that would cast light on the matter. It is this issue of historical nosology, rather than historical epidemiology, that the present study aims to address.

## Materials for study

The problem of determining how viewers in the past subjectively understood medical aspects of a painting or sculpture is a difficult one that usually allows only a highly speculative response. The present study seeks to avoid excessive speculation by considering a special case for which substantial relevant information is available. The analysis will focus on Renaissance images of the Virgin Mary produced by the Florentine sculptor, Francesco di Simone Ferrucci (1437–1493).

The Virgin Mary or Madonna serves as a special case for present purposes because of the strong conviction among Catholics, from the early days of Christianity onward, that she alone among humans was a figure of perfection, exempt from all sin and disease [8]. The audience for devotional images in late fifteenth century Italy expected representations of the Madonna to conform to this understanding of her spiritual and physical perfection.

Francesco di Simone Ferrucci was a leading producer of bas-relief images of the Virgin and Child for this audience. Although not well-known today, he was ‘one of the most prolific and successful sculptors in Florence during the last quarter of the fifteenth century’ [9]. After receiving initial training in Fiesole from his father Simone, who was himself an accomplished sculptor and stonemason, Francesco moved to Florence and absorbed the artistic style then favored in that city. By 1466 he had established his own workshop and was receiving commissions for altars, tombs, and statues in Florentine religious and public buildings. In addition to undertaking these monumental commissions, however, Francesco’s workshop also turned out large numbers of less expensive devotional sculptures for a wide audience of pious believers.

Through his work in other cities, such as Bologna, Urbino, and Rome, Francesco was influential in spreading the Florentine style of sculpture throughout central Italy [10]. Although his reputation today is overshadowed by that of other Quattrocento masters such as Donatello (c. 1386–1466) and Verrocchio (c. 1435–1488), his sculptures are represented in art galleries around the world. This continuing interest in his work by art specialists, and the commercial success he enjoyed during his own lifetime, make him a significant historical figure and a reliable indicator of mainstream artistic conventions of his time.

## Observations

### *Madonna and child*

**Fig. 1:**
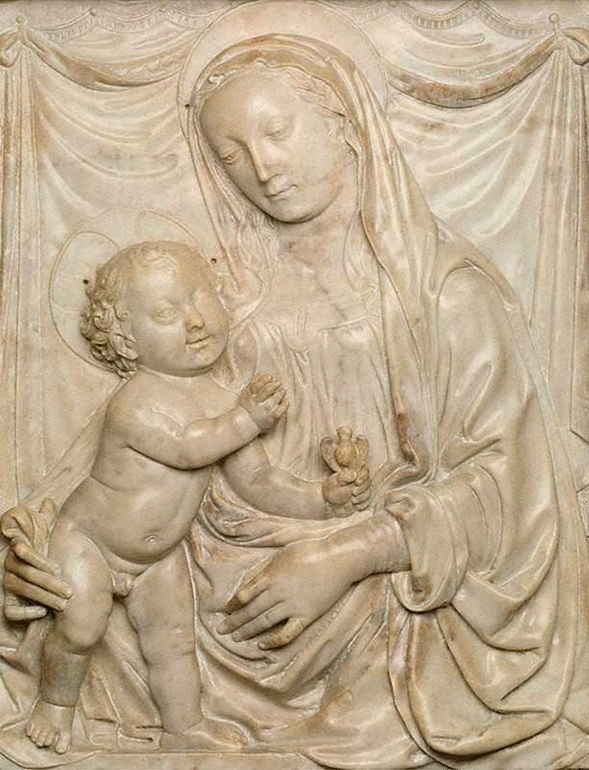
Francesco di Simone Ferrucci, *Madonna and Child*, c. 1480s, Art Gallery of New South Wales, Sydney. Marble bas-relief (90 × 72 × 18 cm with frame) showing apparent camptodactyly in the second and fifth fingers of both the Madonna’s hands. Reproduced with permission from AGNSW, under section 40 of the Copyright Act 1968

Given the cultural expectation that the Virgin should be represented as physically perfect, it is of interest that one can see hand deformities in Ferrucci sculptures such as the marble bas-relief, *Madonna and Child* (Fig. 1), now in the Art Gallery of New South Wales (Sydney).

This work, dated c. 1480s, is attributed by the Art Gallery of New South Wales to Francesco di Simone, but the attribution has been disputed by Pisani, who considers it a ‘high-quality replica’ of a work by Francesco’s younger cousin once removed, Andrea di Piero Ferrucci (1465–1526). Andrea’s version of this relief is located on the funeral monument for Giordano Orsini, which Pisani dates to the period between 1484 and c. 1500 [10, 11].

While the question of attribution is a matter of keen interest to art historians, it is less important for the purposes of the present article. The dates proposed for the two sculptures are approximate only, making it impossible to determine which of these two works preceded the other one. Also Andrea—who received his training from Francesco—is known to have frequently re-used motifs and stylistic features found in his older kinsman’s work [10]. So it is not an easy matter to say which version of the bas-relief is a copy of the other one, or whether they are both copies of some third, now lost, original.

Because of the high demand for devotional sculptures of the Virgin and Child during the fifteenth century in Florence [12], these artworks were often designed to be reproduced in multiple copies [12, 13]. The fact that this happened in the present case strengthens the point being made here, since for devotional objects like the one under consideration, ‘it was the final quality of the work itself that counted, and not the “originality” of its creator’ [9]. Our concern is with the anatomical details of the Madonna’s hands, and if they are of a kind that was frequently reproduced, this confirms that they were in keeping with the artistic tastes and religious expectations of the time.

To a modern eye the young mother’s left hand appears to be deformed with camptodactyly in the proximal interphalangeal (PIP) joint of the second finger and the fifth finger, while her right hand also shows the appearance of this deformity in the mid-joint flexion of the second and fifth fingers. It is possible that her hands were modelled on those of someone who was actually suffering from camptodactyly, but this possibility is unlikely since camptodactyly is an uncommon congenital condition.

Although it does occur in young women, it affects less than 1% of the population today [14, 15]. This statistic may reflect under-reporting because of mild cases being overlooked when the condition ‘is restricted to the fifth finger and does not interfere with the function of the hand’ [16]. But involvement of the second finger, as appears in Fig. 1, is particularly rare [15]. And even if the model did have camptodactyly, the main point at issue here—that camptodactyly was not recognised as pathological in late fifteenth century Italy—would still be supported. The artist would not have copied this model’s hands for his Madonna figure if people in his society regarded them as deformed.

A more likely alternative to deformity in the model is that the Madonna’s hands were sculpted this way to suit the stylistic conventions of the time—‘beautified’ on the artist’s own initiative or at the request of the patron who commissioned the original work. Francesco, like Verrocchio and other contemporary sculptors working in the Florentine style, produced many images of the Madonna with similar hand deformities [10]. This prevalence reinforces the suggestion that mild camptodactyly in an otherwise healthy hand was not considered pathological at the time since in religious terms, as previously noted, the Madonna’s image was expected to show a figure of perfection, and in medical terms the condition is largely asymptomatic and does not interfere with normal functioning unless the contracture is extreme [14].

### *Adoration of the shepherds*

**Fig. 2:**
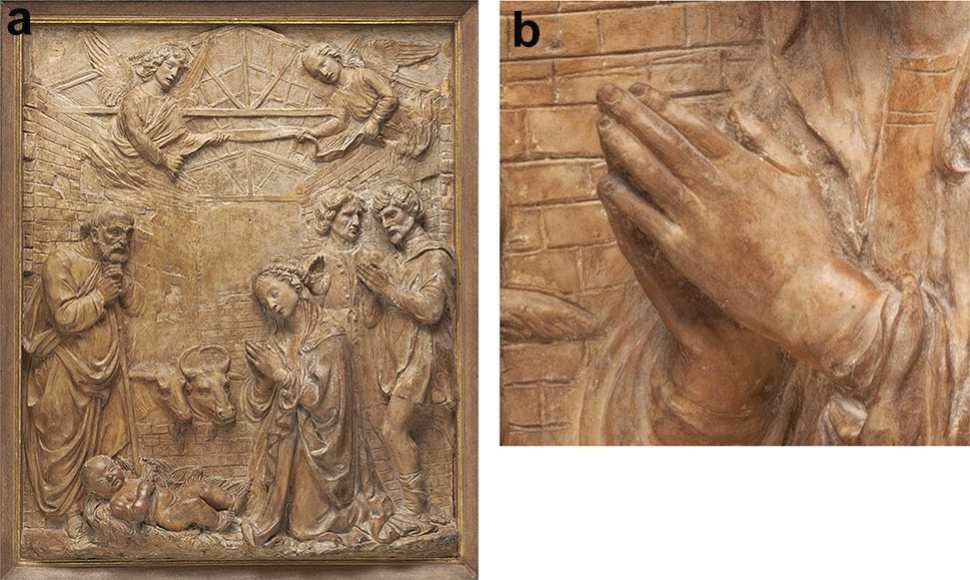
**a** Francesco di Simone Ferrucci, *Adoration of the Shepherds*, c. 1475/1485, National Gallery of Art, Washington, DC. Terracotta bas-relief (132.72 × 102.87 × 11.11 cm with frame) depicting the nativity scene with infant Jesus on a bed of straw on the ground, Virgin Mary kneeling in prayer at the infant’s feet, Joseph standing and leaning on his staff at the infant’s head, two shepherds standing behind Mary, and two angels in the air above the scene holding a banderole between them. Open Access Image, courtesy National Gallery of Art, Washington. **b** Detail of **a** showing apparent boutonniere deformity in the second finger of Virgin Mary’s left hand

Further support for the non-pathological ‘beautification’ hypothesis, involving a visually similar but etiologically different deformity, comes from a terracotta bas-relief by Francesco di Simone in the National Gallery of Art (Washington), showing the* Adoration of the shepherds* (Fig. 2a).

The hands of the elderly St Joseph and the two middle-aged shepherds are normal, but the second finger of the young Madonna’s left hand has what appears to be a mild boutonniere deformity (Fig. 2b).

In this deformity, as in camptodactyly, the PIP joint is flexed, but in addition the distal interphalangeal (DIP) joint is hyperextended. This deformity is usually traumatic or inflammatory in origin rather than congenital [17]. In a normal hand it would not be possible to position the index finger as shown in the sculpture without exerting pressure on the DIP joint, but the hands here are only lightly resting together at the fingertips.

It is significant that there are no hand deformities in the older men, where one might expect to see arthritic or traumatic changes in the hands of a manual laborer like St Joseph the carpenter or peasants like the two shepherds. Instead, it is the young Madonna who shows a hand deformity—a mild one rather than grossly disfiguring, but still inappropriate if it was likely to have been understood as pathological by religious viewers of the time. Hence, on balance, the suggestion of stylistic ‘beautification’ is supported. As a result, boutonniere deformity should be considered a stylistic option for artists of this period, and not necessarily a sign of pathology in the subject portrayed.

## Discussion

The bas-relief sculptures by Francesco di Simone discussed here accurately reproduce the appearance of camptodactyly and boutonniere deformity in figures of the Madonna, even though the young people who served as the original models for these figures are unlikely to have had such pathologies themselves. The sculptures do not provide enough information to support a confident diagnosis of camptodactyly and boutonniere deformity, but for the purposes of this study it is sufficient that they do present the appearance of these conditions. It is the appearance of the Madonna’s hands that Francesco’s fifteenth century audience would be considering when they viewed his sculptures, and the question at issue is whether or not that audience would regard hands with apparent camptodactyly or apparent boutonniere deformity as pathological.

Camptodactyly appears quite often in western European art of the fifteenth and early sixteenth century [16], usually in persons of high social status. Boutonniere deformity, although less prevalent in Renaissance art, can nevertheless be found in the paintings of masters like Lucas Cranach the elder (1472–1553) and Raphael Sanzio (1483–1520). Cranach’s *Three Apostles* in the Queensland Art Gallery (Brisbane), for example, includes a praying apostle whose right hand appears to have a boutonniere deformity in the fourth finger as well as camptodactyly in the fifth finger [18]. Raphael’s *Young Man with an Apple* in the Galleria Palatina (Florence) shows an aristocratic gentleman with apparent boutonniere deformity in the third and fourth fingers of his left hand [19].

One may suspect on the basis of the high social status of aristocrats and religious figures such as apostles, that camptodactyly and boutonniere deformity were not regarded as pathological in the Renaissance period. The presence of these types of deformity in the Madonna’s hand, as depicted in Francesco di Simone’s sculptures, gives strong confirmation to this suspicion because of the religious beliefs of the time regarding the Madonna’s physical perfection.

## Conclusion

The evidence presented here regarding camptodactyly and boutonniere deformity supports the conclusion that the appearance of the hand in mild cases of both these conditions was considered elegant and graceful in Renaissance art, as Johnson suggested for camptodactyly [6], rather than pathological. In the works by Francesco di Simone just examined, this type of appearance lent an aristocratic or queenly air to the representation of the Madonna, who was regarded by believers as the physically perfect ‘queen of heaven’.

These findings extend to the medium of sculpture the results of previous studies showing that certain hand deformities, predominantly camptodactyly and clinodactyly, were frequently used as indicators of high social status in Renaissance paintings [7], and they also add boutonniere deformity to the other conditions already documented. This information must be taken into account in any attempt to consider Renaissance artworks for the purposes of retrospective diagnosis and historical epidemiology. In addition, for the purposes of historical nosology, the present findings show that camptodactyly and boutonniere deformity, at least in mild cases, were not regarded as pathological in late fifteenth century Italy.
